# An intervention to support persons living alone with cognitive impairment in the United States

**DOI:** 10.1002/alz.087620

**Published:** 2025-01-09

**Authors:** Allison Gibson

**Affiliations:** ^1^ St. Louis University, St. Louis, MO USA

## Abstract

**Background:**

Although most individuals experiencing cognitive impairment reside with another, as many as one third may live alone. Such individuals may have difficulty managing their health and wellbeing. Further, they may be more isolated from healthcare and social services. Developing strategies to circumvent premature institutional placement and reduce use of emergency services is vital to keeping healthcare expenditures lower, and also recognizes the needs of individual’s safety, autonomy, and quality‐of‐life.

**Methods:**

This study utilized a single‐arm, non‐randomized trial design. This proof‐of‐concept study aimed to establish feasibility of Services To Age in Your Home (STAY Home) service utilization and care management intervention to improve utilization of healthcare and social support services for persons living alone with cognitive impairment. The intervention implemented one session weekly for twelve weeks through in‐person visits by a team of social workers and student interns of approximately one hour. Potential participants underwent cognitive screenings, the Adapted Informed Consent Questionnaire, and the informed consent process was repeated every 30 days to try to ensure that participants were appropriate to participate. The primary outcome was to increase healthcare and social service utilization. Exploratory outcomes examined social support and biopsychosocial needs.

**Results:**

Of the 12 participants, individuals reported limited, non‐traditional forms of social support. Analyses of the data demonstrate a significant need for this population to link those living alone with cognitive impairment to healthcare and social services (pre‐intervention only 42% had been seen by a social serivce provider in the last year; only 33% had seen a healthcare provider in the last year). Analyses demonstrate the intervention is feasible with high adherence to weekly visits (75%). While the study was not powered to compare secondary outcomes, quality‐of‐life increased for participants from pre‐ to post‐intervention (pre‐intervention x̄ = 2.7; post‐intervention x̄ = 3.3; p‐value = 0.01).

**Conclusion:**

While many participants were willing to engage in the pilot program, additional barriers contributed to utilizing health and social services. Future studies must continue to explore potential solutions for this difficult‐to‐support population. In addition to increasing access to healthcare and support services, participants may also benefit from a medication adherence component to the intervention.

